# Variability in Bulb Organosulfur Compounds, Sugars, Phenolics, and Pyruvate among Greek Garlic Genotypes: Association with Antioxidant Properties

**DOI:** 10.3390/antiox9100967

**Published:** 2020-10-09

**Authors:** Ioanna Avgeri, Konstantina Zeliou, Spyridon A. Petropoulos, Penelope J. Bebeli, Vasileios Papasotiropoulos, Fotini N. Lamari

**Affiliations:** 1 Laboratory of Pharmacognosy and Natural Products, Department of Pharmacy, University of Patras, 26 504 Patras, Greece; augeriioanna@gmail.com (I.A.); konzeliou@upatras.gr (K.Z.); 2 Laboratory of Vegetable Production, Department of Agriculture Crop Production and Rural Environment, University of Thessaly, Fytokou, 38 446 Volos, Greece; spetropoulos@uth.gr; 3 Laboratory of Plant Breeding and Biometry, Department of Crop Science, Agricultural University of Athens, Iera Odos 75, 118 55 Athens, Greece; bebeli@aua.gr; 4 Department of Agriculture, University of Patras, Theodoropoulou Terma, 27 200 Amaliada, Greece; vpapasot@upatras.gr

**Keywords:** Alliaceae, radical scavenging activity, crop landraces, ultrasound extraction, pyruvic acid, carbohydrates, vinyldithiins, chemical diversity, gas chromatography

## Abstract

In order to assess the diversity of Greek garlic (*Allium sativum* L.) landraces, 34 genotypes including commercial ones were grown in the same field and their content in organosulfur compounds, pyruvate, total sugars, and total phenolics, alongside antioxidant capacity, was determined. The organosulfur compounds were studied by Gas Chromatography–Mass Spectrometry (GC–MS) after ultrasound-assisted extraction in ethyl acetate, identifying 2-vinyl-4H-1,3-dithiin and 3-vinyl-4H-1,2-dithiin as the predominant compounds, albeit in different ratios among genotypes. The bioactivity and the polar metabolites were determined in hydromethanolic extracts. A great variability was revealed, and nearly one-third of landraces had higher concentration of compounds determining bioactivity and organoleptic traits than the imported ones. We recorded strong correlations between pyruvate and total organosulfur compounds, and between antioxidant capacity and phenolics. In conclusion, chemical characterization revealed great genotype-dependent variation in the antioxidant properties and the chemical characters, identifying specific landraces with superior traits and nutritional and pharmaceutical value.

## 1. Introduction

Common garlic (*Allium sativum* L., family Alliaceae) is the second most widely consumed bulb crop and one of the most cultivated bulb vegetables in Greece and worldwide, with an annual production of 28,494,130 tons and a total harvested area of 1,546,741 hectares [1,2]. It is consumed raw, cooked, or as an ingredient of herbal medicinal products and food supplements [3,4]. Garlic is considered effective and safe for the prevention and treatment of cardiovascular and other metabolic diseases, such as atherosclerosis, hyperlipidemia, thrombosis, hypertension, and diabetes; it also possesses antifungal, antibacterial, and antiviral properties and regulates blood sugar levels [5]. Among other biological mechanisms mediated by its components, garlic extracts also present significant in vitro and in vivo antioxidant properties [3].

Raw garlic bulbs contain mostly water, carbohydrates, and proteins but also trace elements and vitamins [1]. The main bioactive compounds are saponins, flavonoids, organic acids, and various organosulfur compounds [3]. The latter are present in intact bulbs as peptides, like γ-glutamyl-S-alk(en)yl-L-cysteine, and sulfoxides of S-alk(en)ylo-L-cysteine, like alliin, which is the predominant cysteine derivative. This compound is metabolized to allicin by the enzyme alliinase, when the bulb is crushed, also producing ammonia and pyruvic acid. Allicin and other sulfoxides may undergo many transformations both in vitro and in vivo, resulting in a wide variety of organosulfur volatiles, which are responsible for the flavor and aroma, as well as for most of the beneficial health effects of garlic [6,7]. Although many differences in the bioaccessibility and bioactivity of those compounds have been recorded so far, phenolics and saponins may also contribute to the antioxidant and anti-inflammatory properties of garlic, whereas polysaccharides (>85% fructose) have exhibited immunomodulatory effects [3,7]. Furthermore, the chemical composition and organoleptic characteristics of garlic are influenced by the genotype, the cultivation/environmental conditions, and the processing methods (temperature, pH, solvent) [8].

Garlic is a completely sterile diploid species, which has been clonally propagated for centuries [9]. Over time, cultivated garlic clones or clonal lineages have been established through domestication in several cultivation centers. These distinct genotypes have gained adaptation to different agroclimatic conditions and various ecotypes, exhibiting large-scale phenotypic diversity and variation in several traits [9]. Variation among garlic genotypes is the basis for breeding new varieties with superior traits. In this context, there is considerable interest for local genotypes (landraces and/or farmers’ varieties) with respect to their content of bioactive compounds and the antioxidant properties of its cloves [10,11,12].

Crop landraces comprise an important part of agricultural biodiversity. Landraces are variable populations, genetically diverse, lacking “formal” crop improvement. They constitute an invaluable genetic pool due to their characteristics including local adaptation, resilience to biotic and abiotic conditions, and considerable organoleptic traits and nutritional value [13]. During the last years, they have been displaced by more productive and uniform improved varieties and hybrids, a trend which has led to a reduction of the crops’ genetic base, and subsequently to genetic erosion, and to an increased threat of genetic vulnerability. Recently, due to the increased demand for natural, local, and high-quality products produced by traditional and environmentally friendly practices, landraces have been rediscovered as a source of value-added foods [14].

The phenotypic diversity and nutritional value of certain Greek garlic genotypes have recently been reported by our groups [11,12,15]. The aim of the present study was to determine the main bioactive compounds and to evaluate in vitro the antioxidant properties of Greek garlic germplasm; organosulfur compounds were determined for the first time and ultrasound-assisted extraction was adopted for the study of both volatiles and polar ingredients. For that purpose, we cultivated 34 garlic genotypes, including Greek landraces and commercial cultivars, under the same conditions (same location and cultivation practices). It was expected that the study of many local and imported garlic genotypes would reveal genotype-dependent diversity in chemical characters and antioxidant properties and contribute to the exploitation and valorization of this valuable genetic material.

## 2. Materials and Methods

### 2.1. Plant Material

Thirty-four garlic genotypes, including 29 local and commercialized landraces and 4 commercial cultivars, were examined in the present study. The geographical coordinates of the genotypes’ collection sites are presented in Table 1. The garlic genotypes were planted and cultivated in the experimental field of Kavasila, Ileia Regional Unit (37°52′ Ν, 21°17′ Ε) during the growing period 2016–2017 (all the accessions were planted on 5 December 2016 and harvested on 15 June 2017), as previously described [15].

### 2.2. Preparation of Extracts

Cloves of fresh garlic bulbs were separated and skinned; 10 g of each accession were weighed and ground to a paste with a mortar and a pestle. The obtained garlic paste was subjected twice to ultrasound-assisted extraction (UAE) in an ultrasound bath (40 kHz, ISOLAB Laborgeräte GmbH, Wertheim, Germany) for 30 min with 60 mL ethyl acetate each time. The ethyl acetate extracts were collected and extracted further with water, while the remaining garlic paste was extracted with 100 mL methanol:water (50/50, *v*/*v*) under stirring for 24 h. The aqueous phase and the hydromethanolic extract were pooled and lyophilized (polar extract), while the ethyl acetate extract (nonpolar extract) was concentrated with nitrogen. The extracts were stored at −20 °C until further use.

### 2.3. Determination of Dry Weight 

Dry weight (D.W.) was calculated by heating approximately 10 g of fresh sample (5–10 cloves) in preweighed porcelains at 105 ± 2 °C for 22–24 h, until constant weight. Samples were cooled down for 30 min in laboratory desiccators containing silica gel and then weighed.

### 2.4. GC–MS Analysis of Volatiles in Nonpolar Extracts 

Analysis was performed by GC–MS on Agilent 6890N GC apparatus coupled to an Agilent 5975 B mass spectrometer (Agilent Technologies, CA, USA), with a nonpolar column HP-5MS (30.0 m × 250.00 μm, film thickness 0.25 μm), with electron impact ionization energy at 70 eV. Helium was used as a carrier gas at 1.0 mL/min flow rate. Injection volume was 1 μL in splitless mode; scan range was 50–1050 *m*/*z*. Injector temperature was set at 300 °C, and source temperature at 230 °C. Solvent delay was set at 3 min, initial oven temperature was 50 °C and then was ramped at 1 °C min^−1^ to 61 °C, remained at 61 °C for 4 min, ramped at 1 °C min^−1^ to 115 °C, and then at 2 °C min^−1^ to 191 °C and at 15 °C min^−1^ to 281 °C, remained at 281 °C for 3 min, and finally ramped at 25 °C min^−1^ to 300 °C.

Tentative identification was performed by examination and comparison to the literature of their MS spectra and retention indices (AI), using the Van den Dool and Kratz equation based on a series of linear alkanes, C8-C20 and C21-C40 [16]. Octane was used as both an internal and external standard. Concentration (from duplicate analyses) was determined as n-octane equivalents through the equation

*y* = 1.6199*x* + 0.0244 (*R*^2^ = 0.982)

where *y* = μg n-octane /mL and *x* = response factor of the analytes (i.e., the ratio of peak area of each analyte to that of the internal standard at the concentration of 1.20 g L^−1^); the calibration curve was established with seven different n-octane concentrations (0.15, 0.30, 0.60, 1.20, 1.60, 2.00, and 2.50 g L^−1^). The coefficient of variation of the analyses never exceeded 14.8%. Detection level was set at 0.1% of total peak area. Peaks were quantified only if their response factor was higher than 0.025. 

### 2.5. Determination of Pyruvic Acid, Total Sugars, Total Phenolics, and Antioxidant Activity of Hydromethanolic Extracts

Pyruvic acid, total phenolics, total sugars content, and antioxidant capacity were measured in the dry aqueous methanolic extracts (twice in triplicates). All methods except for that of hydrogen peroxide scavenging were adapted for 96-well plates and the absorbance was measured in a UV/vis microplate reader (Sunrise, Tecan, Männedorf, Switzerland). 

Pyruvic acid concentration was estimated as earlier described [17]. Briefly, 10 µL of sample (concentrations 2.5, 5, and 10 g dry extract L^−1^) or standard (sodium pyruvate) was added to 90 µL of formaldehyde-2,4-dinitrophenylhydrazone (DNPH) (0.63 mM DNPH reagent in 0.5 mol L^−1^ HCl), and incubated for 30 min at 25 °C. Afterwards, 50 µL of KOH (5 mol L^−1^) was added and incubated for 30 min at 37 °C. Absorbance was measured at 540 nm and the concentration is expressed as μmol of sodium pyruvate per 100 g of fresh weight (F.W.) according to the equation *y* = 0.161*x* + 0.006 (*R*^2^ = 0.999) produced by sodium pyruvate concentrations 0.25, 0.50, 1.00, 2.00, and 4.00 mmol L^−1^. 

Total sugars were determined by the anthrone method [18,19]. Forty μL of samples (50, 80, and 100 mg dry extract L^−1^) or standard sucrose (0.015, 0.030, 0.060, 0.120, 0.240, and 0.480 g L^−1^) or blank were cooled at 4 °C for 15 min and then were mixed with 100 μL of freshly prepared anthrone reagent (2 g L^−1^ in concentrated sulfuric acid). After 3 min in a water bath at 92 °C, the microplate was immersed in a water bath at 25 °C for 5 min and then was placed in an oven at 45 °C for 15 min. Absorbance was measured at 620 nm and concentration is expressed as mg sucrose equivalents per 100 g of F.W., according to the equation *y* = 0.409*x* − 0.002 (*R*^2^ = 0.999).

Total phenolic content was determined with the Folin–Ciocalteu reagent method at 620 nm [19]. In brief, samples (20 μL of 3.5, 5.0, and 8.0 g dry extract L^−1^) or the respective blanks, Folin–Ciocalteu reagent 10% *w*/*v* (40 μL), and a solution of 7.5% *w*/*w* sodium carbonate (160 μL) were mixed and left in the dark for 45 min. The total phenolic content is expressed as mg of gallic acid equivalents (GAE) per 100 g of F.W. with the calibration curve *y* = 0.006*x* − 0.012, *R*^2^ = 0.999 generated by gallic acid concentrations 3.13, 6.25, 12.50, 25.00, and 50.00 mg L^−1^.

The antioxidant activity of the dry methanolic extracts was evaluated with two different assays: the ferric reducing antioxidant power (FRAP) and the hydrogen peroxide (H_2_O_2_) scavenging methods. The FRAP method measures the ability of antioxidants to reduce the [Fe(TPTZ)_2_]^3+^ to [Fe(TPTZ)_2_]^2+^ [20]. In detail, 80 μL of FRAP solution (15 mL of a solution of 10 mM TPTZ [2,4,6-tri(2–pyridyl)–s–triazine] in 40 mM HCl, 15 mL of 20 mM FeCl_3_.6H_2_O, and 75 mL of 300 mM acetate buffer solution, pH 3.6) was mixed with 55 μL of acetate buffer and 40 μL extract (5 to 10 g dry extract L^−1^) or standard (FeSO_4_.7H_2_O) and incubated at room temperature (RT) for 5 min. Absorbance was measured at 592 nm and the results are expressed as μmol FeSO_4_ per 100 g of F.W., with the aid of the calibration curve *y* = 3.652*x* − 0.187 (*R*^2^ = 0.997) produced by FeSO_4_.7H_2_O concentrations 0.05, 0.10, 0.15, 0.20, 0.30, and 0.40 mmol L^−1^. The hydrogen peroxide (H_2_O_2_) scavenging method estimates the scavenging activity towards H_2_O_2_ and superoxide radical [21]. For this purpose, an H_2_O_2_ (43 mM) solution was prepared in phosphate buffer (0.1 M, pH 7.4). Extracts (4 g dry extract L^−1^) as well as ascorbic acid (0.1 to 0.8 g L^−1^) in 3.4 mL phosphate buffer were added to 0.6 mL of H_2_O_2_ solution. The percentage of H_2_O_2_ scavenging of ascorbic acid and extracts was calculated by measuring the absorbance at 230 nm, subtracting that of their respective blanks (extracts only), and comparing to that of H_2_O_2_ alone. H_2_O_2_ scavenging effect is expressed as g ascorbic acid equivalents/100 g F.W. 

### 2.6. Statistical Analysis

Spearman’s correlation was performed for all variable pairs at a significance level of 95% (α = 0.05) and *r* > 0.90, *r* > 0.70, *r* > 0.50, *r* > 0.30 are interpreted as very high, high, moderate, and low coefficients, respectively. The SPSS software version 25.0 (IBM Corp., Armonk, NY, USA) was used for data analysis. Value standardization and heatmap were performed with PRISM 8 (Graph Pad, San Diego, CA, USA).

## 3. Results and Discussion

### 3.1. Extraction Protocol, Volatiles, and Pyruvic Acid

UAE was used for the extraction of garlic volatiles based on the methodology earlier described [22]; in that study, the authors demonstrated that UAE diminishes the danger of thermal decomposition of sensitive aroma compounds. In the present study, a slight modification in the extraction protocol was applied, that is, the garlic homogenate was firstly extracted with ethyl acetate and then with aqueous methanol. Moreover, the extractions of the organic solvent phase were performed only with water to collect all aqueous phases and then to determine the polar ingredients and the antioxidant properties. As a result, with the above described pretreatment modification, we managed to determine both polar and nonpolar ingredients with the same amount of plant material.

The yield of ethyl acetate extract varied among the genotypes from 0.04% volume/weight (*v*/*w*) (AS06) to 0.30% *v*/*w* (AS30) as presented in Table 2. The GC–MS analysis of the ethyl acetate extract revealed the identity of 18 volatiles, which are organosulfur compounds and alkanes (Table 3). Concerning the organosulfur compounds, the acyclic monosulfide ethyl vinyl sulfide (peak 1) has been earlier reported [22], whereas diallyl sulfide (peak 2) is a common acyclic sulfide that has been reported by many research groups [22,23,24,25,26,27]. Other common acyclic disulfides and trisulfides are methyl allyl disulfide (MADS; peak 5), diallyl disulfide (DDS; peak 9), 1-propenyl allyl disulfide (peak 11), allyl methyl trisulfide (MATS; peak 12), and diallyl trisulfide (DATS; peak 19) [22,23,24,25,26,27,28,29]. We also identified the following cyclic disulfides: 3-vinyl-4H-1,2-dithiin (3-VDT; peak 14) (a common one [6]), 3H-1,2-dithiole (peak 6) [25,27], and 3-dithiane (or 3,4-dihydro-1,2-dithiin) (peak 10). The latter has been earlier wrongly ascribed as trithiacyclohexene, whereas we identified only one cyclic trisulfide (4H-1,2,3-trithiine; peak 15) [25]. With regard to cyclic sulfides, 2-vinyl-4H-1,3-dithiin (2-VDT; peak 17), which is a common garlic ingredient, and 2-vinyl-1,3-dithiane (peak 18) were also determined in the extracts. Lastly, we detected the presence of the cyclic thione (3-methyl-2-cyclopentene-1-thione; peak 7) and we suggest that the closely eluting compound (peak 8) is a cyclic thiol (4-methylcyclopenta-1,3-diene-1-thiol) based on its mass spectrum (Table 3).

Among those organosulfur compounds, 2-VDT, 3-VDT, and DDS were detected and quantified in all genotypes examined, while 3-VDT and 2-VDT were the predominant compounds (45.7 ± 7.5% and 30.9 ± 10.2%, respectively) (Table 2). An organosulfur compound that could not be fully identified (compound 13) was detected in all tested genotypes in relatively high amounts (6.3 ± 3.0%); it reached nearly 15.0% of organosulfur compounds in AS01 and AS10 (Table 2). The ratio of 3-VDT to 2-VDT in most genotypes was about 1, except for AS04, AS05, AS08, AS10, AS25, AS31, AS35, and AS36 genotypes in which the ratio ranged from 3.4 to 3.9. Our results contribute to the quest for garlic genotypes and processing methods which can provide high 3-VDT content [30,31]. Since 3-VDT is more lipophilic and inhibits the differentiation of preadipocytes, it can be a beneficial agent against obesity, along with its other beneficial antioxidant and cholesterol-lowering properties [32]. Based on our results, genotypes AS36 and AS25 could be good candidates for that purpose. 

The detection of vinyl-dithiins in most of the tested genotypes is in accordance with studies performed in raw garlic where the plant material is not subjected to high temperatures. The cyclic dithiins are presumed to be the first products of allicin transformation, while acyclic compounds are produced during the thermal degradation of cyclic dithiins [22]. Indeed, other researchers who used various distillation methods for the extraction of garlic volatiles also found that organosulfur compounds such as DDS, diallyl trisulfide, and methyl allyl trisulfide were among the four most abundant ones [4,23,28,29]. In our study, DDS was also an important volatile constituent detected in percentages ranging from 1.81 to 8.55% (4.34 ± 1.47%). This finding is in agreement with earlier observations that only DDS was present in extracts obtained under mild conditions and not with thermal treatment [28]. 

Even if a part of allicin is converted during GC analysis to divinylthiins and other organosulfur compounds [22], the differences described above (e.g., the ratio of 3-VDT to 2-VDT) among the genotypes indicate that this process is highly complex and matrix-dependent. Recent studies reported that organosulfur compounds are also formed nonenzymatically in the aqueous environment of raw garlic at room temperature and thus are naturally occurring and are responsible for its distinct aroma [33]. In particular, 2-VDT has the highest flavor dilution factor among other volatiles and thus determines aroma of fresh garlic samples [6]. Therefore, GC profiling gives information not only on the different quantities of alliin and other γ-glutamylalk(en)ylcysteine precursors, but also on the aroma-responsible transformation products which are naturally occurring in the untreated (raw) plant material, while any observed differences are also genotype-dependent. 

**Figure 1:**
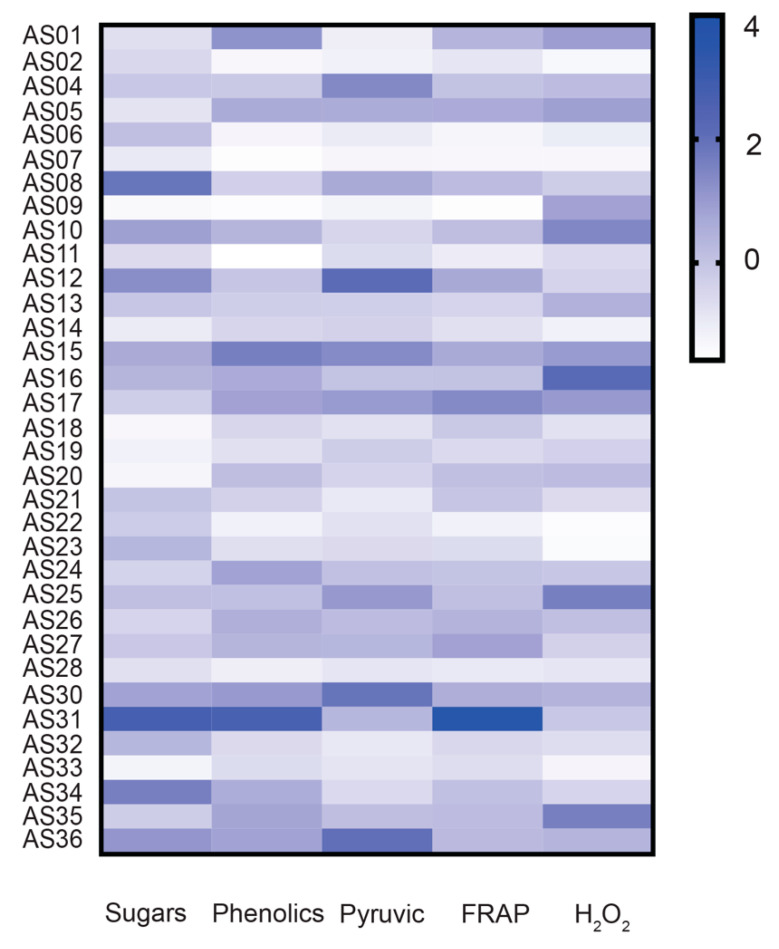
Heat map showing the variation of total sugars, phenolics, and pyruvate, and the antioxidant properties by FRAP and H_2_O_2_ scavenging assays in 34 garlic genotypes. Standardized values (z-scores) of mean values are depicted with color scale (from light to intense blue). Raw data are provided in Table 4.

In a recent study, pyruvate constituted up to 61% of total organic acids in garlic [11]. Determination of pyruvate has been used for the indirect estimation of allicin in fresh raw garlic since it is the by-product of alliin transformation to allicin [34]. In the present study, the pyruvic acid content in the hydromethanolic extracts varied greatly among genotypes (Figure 1, Table 4) from 369.45 (AS07) to 7246.69 (AS12) μmol sodium pyruvate equivalents per 100 g of F.W. 

A moderate correlation was observed between pyruvic acid content and nonpolar extract (ethyl acetate) yield (*r =* 0.690, *p* < 0.01). In contrast, a high correlation was observed between the pyruvic acid content and the total organosulfur volatiles content detected by GC-MS (*r =* 0.817, *p* < 0.01), as well as between the ethyl acetate extract yield and the total organosulfur volatiles content (*r =* 0.801, *p* < 0.01). These results confirm earlier studies showing that pyruvate levels are significantly and positively correlated with individual and total organosulfur content in garlic [34,35].

Previously, a positive association between pyruvate levels and flavor (pungency) intensity [36] and antiplatelet activity [35] has been reported. In the present study, the great variation in pyruvate levels (varying nearly 15-fold between the genotypes with the lowest and the highest content) could allow the selection of mild and pungent garlic genotypes, as well as genotypes with high functional value, for selection in future breeding programs. Thus, the landraces AS04, AS12, AS15, AS17, AS25, and AS36 presented the highest levels of pyruvic acid and total organosulfur compounds (higher than all the genotypes tested) and therefore could be characterized by the most intense flavor and taste. 

### 3.2. Total Sugars, Phenolics, and Antioxidant Activity 

Due to the complexity of redox mechanisms in humans, there is not a single in vitro assay for the estimation of total antioxidant capacity of food but plenty of them which employ different mechanisms and probably estimate the activity of different chemical compounds. In this study, two complementary assays were used for the in vitro assessment of antioxidant capacity, one hydrogen atom transfer assay (H_2_O_2_ scavenging) at a physiological pH and one single electron transfer assay (FRAP) at a low pH (3.6). In parallel, the content of total phenolics and sugars was estimated. As shown in Figure 1 and Table 4, the concentration of total sugars and phenolics, as well as the antioxidant activity values, varied greatly among the tested garlic genotypes. According to previous studies [12,37], the variation in total phenolic compounds content could be attributed to the growing location as well as to genotypic differences and the cultivation practices. Herein, considering that all the tested genotypes were cultivated at the same location and under the same cultivation practices, any variation found could be attributed to differences in the genetic background of the genotypes.

To investigate the relationships among the determined compounds and the antioxidant capacity of the tested garlic genotypes, a correlation analysis was performed, and the results are presented in Table 5. A strong positive correlation between total phenolics and FRAP assay was observed. Accordingly, scavenging activity towards H_2_O_2_ was moderately correlated with total phenolics and the FRAP assay. Similarly, pyruvic acid content was strongly correlated with the FRAP assay and moderately correlated with sugars, total phenolics, and the H_2_O_2_ scavenging activity.

The total phenolic concentration in the *Allium* genus is possibly correlated with its strong antioxidant, anti-inflammatory, and anticancer properties [38]. Furthermore, the total phenolics content in garlic was positively and strongly correlated with antioxidant capacity regardless of their individual phenolic compounds’ composition [10,38]. In the present study, there is strong evidence of such correlation between the total phenolic compounds content and the antioxidant properties estimated by the FRAP and H_2_O_2_ scavenging activity assays.

## 4. Conclusions

The chemical characterization of garlic genotypes performed in the present study revealed important correlations among the content of volatiles, polar constituents (total sugars, total phenolics, pyruvate), and antioxidant properties enabling us to identify local Greek landraces with superior characteristics which could be further exploited. It has been earlier demonstrated that the successive accumulation of somatic mutations in ancestral cultivars combined with clonal propagation leads to heterogeneity of cultivated clones. This could be the case for the dissimilarities observed in landraces obtained from the same or nearby regions in our study. Another explanation could be the exchange of germplasm between farmers and the deliberate introduction of genetic material from remote origin with different organoleptic traits.

Nearly one-third of the tested genotypes had higher pyruvate and total organosulfur concentration than the imported cultivated varieties. Among the tested genotypes, AS12 and AS36 had the highest pyruvate content and high concentrations of total sugars, AS15, AS36, and AS25 were the most abundant in organosulfur volatiles, while AS15 had the best overall performance in all the measurements. On the other hand, the high prevalence of superior characteristics in landraces originating from the Ionian islands, that is, Kefalonia and Lefkada and the neighboring Peloponnese areas of Arkadia and Messinia prompts us to further valorize these localities for the identification of promising garlic landraces. The selection of superior genotypes could be used in breeding efforts to produce distinct garlic varieties of specific origin with high content of bioactive ingredients and great nutritional, nutraceutical, and pharmaceutical value.

## Figures and Tables

**Table 1:** Geographical distribution and collection sites of the garlic genotypes.

Accessions	Collection Site	Prefecture	Latitude	Longitude	Altitude (m)
**Region of Ionian Islands**					
AS01	Saint Petros	Lefkada	38°40′ Ν	20°36′ Ε	328
AS05	Κarya	Lefkada	38°45′ Ν	20°38′ Ε	510
AS06	Katouna	Lefkada	38°46′ N	20°42′ Ε	165
AS08	Manasi	Lefkada	38°41′ Ν	20°36′ Ε	557
AS12	Κefalonia	Kefalonia	38°17′ Ν	20°31′ Ε	500
AS30	Saint Theodoros	Kefalonia	38°11′ Ν	20°28′ Ε	2
**Region of Peloponnese**					
AS04	Polichni	Messinia	37°16′ N	21°56′ Ε	432
AS11	Tsoureki	Messinia	37°19′ Ν	21°57′ Ε	467
AS13	Andania	Messinia	37°15′ Ν	21°59′ Ε	85
AS15	Altomira	Messinia	36°58′ Ν	22°13′ Ε	827
AS23	Kakaletri	Messinia	37°24′ Ν	22°55′ Ε	607
AS28	Kitries	Messinia	36°55′ Ν	22°08′ Ε	3
AS32	Megali Mantineia	Messinia	36°57′ Ν	22°09′ Ε	207
AS33	Kato Doloi	Messinia	36°93′ Ν	22°17′ Ε	315
AS07	Tripoli	Arkadia	37°30′ N	22°22′ Ε	662
AS17	Mavriki	Arkadia	37°23′ Ν	22°27′ Ε	950
AS19	Lithovouni	Arkadia	37°28′ Ν	22°27′ Ε	676
AS21	Stadio Tripoleos	Arkadia	37°27′ N	22°26′ Ε	675
AS35	Manthurea	Arkadia	37°24′ Ν	22°23′ Ε	750
AS36	Mavriki	Arkadia	37°23′ Ν	22°27′ Ε	950
AS24	Dermatianika	Lakonia	36°54′ Ν	23°02′ Ε	35
AS27	Neapoli	Lakonia	36°30′ Ν	23°03′ Ε	10
**Region of Epirus**					
AS09	Vrysoula	Ioannina	39°40′ Ν	20°32′ Ε	220
**Region of Central Greece**					
AS10	Trachy, Skyros Isl.	Evia	38°57′ Ν	24°30′ Ε	10
**Region of Thessaly**					
AS18	Rizomylos	Magnesia	39°25′ Ν	23°38′ Ε	62
**Region of Eastern Macedonia and Thrace**					
AS02	Nea Vyssa	Evros	41°35′ Ν	26°32′ Ε	31
AS14	Komotini	Rodopi	41°05′ Ν	25°24′ Ε	42
**Region of the South Aegean**					
AS25	Mesa Vouni, Andros Isl.	Cyclades	37°47′ Ν	24°55′ Ε	585
AS34	Milos Isl.	Cyclades	36°40′ Ν	24°23′ Ε	153
**Imported Genotypes**					
	Name	Country			
AS16 ^2^	Gardos	Spain			
AS26 ^3a^	Ajo Morado de Las Pedroñeras	Spain			
AS31 ^3b^	Ajo Morado de Las Pedroñeras	Spain			
AS20 ^1^	Kineziko	China			
AS22 ^1^	Kineziko	China			

^1^ Variety: commercial variety from China; ^2^ Variety (Gardós): commercial variety coming from Spain; ^3a,b^ Ajo Morado de Las Pedroñeras PGI: traditional variety from Spain obtained from different garlic providers.

**Table 2:** Ethyl acetate extract yield (% *v*/*w*) and mean concentration (mg per 100 g of fresh weight) of volatiles determined in the ethyl acetate extracts of the garlic genotypes ^1^.

		1	2	3	4	5	6	7	8	9	10	11	12	13	14	15	16	17	18	19	
	% *v*/*w* Extract Yield	EVS	DS	m-xylene	o-xylene	MADS	3H-1,2-dithiole	3-methyl-2-cyclopentene-1-thione	4-methyl cyclopenta-1,3-diene-1-thiol	DDS	3-dithiane	allyl-prop-1-enyl disulfide	MATS	Unknown C_5_H_10_S_2_	3-VDT	4H-1,2,3- trithiine	1-dodecene	2-VDT	2-vinyl-1,3- dithiane	DATS	Total Identified Organosulfur Compounds^2^
AS01	0.22	n.d.	0.049	n.d.	n.d.	0.179	n.d.	n.d.	0.119	0.086	0.024	n.d.	n.d.	0.684	1.499	0.021	n.d.	1.199	n.d.	n.q.	4.332
AS02	0.08	n.q.	n.q.	n.d.	n.d.	n.q.	0.138	n.d.	n.q.	0.088	n.d.	n.q.	n.d.	0.307	1.350	0.024	0.037	0.911	n.q.	n.d.	2.820
AS04	0.22	0.786	0.961	n.d.	n.d.	3.011	6.598	1.165	n.d.	3.617	0.914	0.031	0.462	6.898	43.714	3.956	n.d.	11.377	0.241	0.076	89.472
AS05	0.09	0.293	0.339	0.051	n.d.	0.302	2.071	0.275	n.d.	1.316	0.056	0.043	0.013	2.727	14.812	0.464	1.236	3.794	0.109	0.042	27.188
AS06	0.04	n.q.	n.q.	n.d.	n.d.	0.031	0.064	n.d.	n.q.	0.106	n.q.	0.029	n.q.	0.005	0.564	0.027	n.d.	0.505	0.017	n.q.	1.458
AS07	0.06	0.014	0.038	n.d.	n.d.	0.046	0.285	n.d.	n.q.	0.402	n.q.	0.083	n.d.	0.563	2.533	0.129	0.106	2.374	n.q.	0.019	6.680
AS08	0.12	0.287	0.302	0.507	0.265	0.532	2.706	0.295	n.d.	1.575	0.096	0.156	0.054	3.551	16.952	1.001	0.684	4.621	0.132	0.095	33.386
AS09	0.05	0.044	0.077	n.d.	n.d.	0.148	0.496	0.038	n.d.	0.509	0.030	0.096	n.q.	0.886	4.507	0.199	0.037	3.078	0.011	0.040	10.585
AS10	0.11	0.293	0.319	0.605	0.356	0.201	2.152	0.295	n.d.	1.525	0.017	0.113	0.077	4.122	16.521	0.830	1.382	4.860	0.172	0.050	32.125
AS11	0.07	n.d.	0.153	0.178	0.072	0.048	0.936	0.090	n.d.	0.876	n.d.	0.158	n.d.	1.433	9.993	0.301	1.109	7.834	0.042	0.080	21.860
AS12	0.15	0.753	0.688	0.249	0.171	1.027	3.948	0.624	n.d.	3.313	0.228	0.211	0.067	4.589	38.781	1.934	0.425	26.223	0.166	0.310	84.759
AS13	0.12	0.379	0.190	n.d.	n.d.	0.254	1.731	0.060	0.809	1.415	n.d.	0.245	n.d.	1.251	26.685	0.873	1.849	25.831	n.d.	0.254	60.796
AS14	0.08	n.q.	0.100	0.085	0.065	0.046	0.762	n.d.	0.008	0.596	n.q.	n.d.	n.d.	0.712	4.026	0.517	0.808	4.253	0.019	0.019	11.281
AS15	0.26	0.600	0.490	0.336	0.398	1.134	4.648	n.d.	0.647	4.498	0.310	1.042	n.d.	3.905	91.408	2.455	2.711	66.743	0.287	0.596	181.838
AS16	0.11	0.040	0.290	0.118	0.077	0.203	2.447	n.d.	0.080	1.493	0.019	n.q.	n.q.	2.113	8.995	0.970	1.224	8.268	n.d.	0.210	25.698
AS17	0.16	0.517	0.278	n.d.	n.d.	0.567	3.170	n.d.	0.268	2.233	0.158	0.287	n.d.	2.897	45.424	1.438	1.559	39.238	0.363	0.277	98.599
AS18	0.10	0.718	0.292	n.d.	n.d.	0.607	2.479	n.d.	0.155	2.598	0.077	1.319	n.d.	1.763	18.781	1.166	1.732	15.348	0.404	0.504	47.301
AS19	0.19	0.356	0.414	0.104	0.070	0.526	3.089	n.d.	0.156	2.386	0.076	0.148	n.d.	3.460	22.746	1.497	2.501	20.609	0.168	0.234	57.156
AS20	0.12	n.q.	n.d.	0.188	0.128	0.143	0.641	n.d.	0.038	0.580	0.024	0.072	n.d.	0.840	14.353	0.258	2.093	14.305	0.074	0.004	32.025
AS21	0.08	0.004	n.q.	n.d.	n.d.	0.069	0.351	n.d.	n.q.	0.206	n.d.	n.q.	n.q.	0.425	2.663	0.143	n.d.	2.096	n.q.	n.d.	6.303
AS22	0.11	0.093	n.q.	0.247	0.158	0.033	1.022	n.d.	0.032	0.978	n.d.	0.048	n.d.	0.782	9.028	0.400	1.645	10.531	n.d.	0.074	23.153
AS23	0.09	0.157	0.168	0.012	0.016	0.087	1.032	n.d.	0.041	1.018	n.d.	0.118	n.d.	0.889	9.656	0.464	1.432	8.965	n.d.	0.105	22.992
AS24	0.08	0.106	n.q.	n.q.	n.d.	0.262	n.d.	n.d.	n.q.	0.567	0.037	0.467	n.q.	0.319	2.610	0.263	n.d.	2.860	0.005	0.019	7.743
AS25	0.28	1.499	1.057	n.d.	n.d.	1.600	8.192	1.272	n.d.	5.086	0.402	0.210	0.170	9.899	75.042	4.783	n.d.	22.327	0.456	0.192	135.787
AS26	0.20	0.480	0.520	0.163	0.200	0.684	3.246	n.d.	0.377	2.717	0.143	0.039	n.q.	4.287	29.137	1.745	1.880	21.925	0.117	0.259	67.263
AS27	0.17	0.594	0.439	0.104	0.057	0.539	2.376	n.d.	0.119	2.039	0.056	0.182	n.q.	2.413	16.316	1.054	0.943	15.834	0.020	0.105	43.188
AS28	0.07	0.077	0.025	n.d.	n.d.	n.q	0.492	n.d.	n.q.	0.283	n.d.	0.015	n.d.	0.325	3.616	0.238	n.d.	1.956	n.q.	n.q.	7.080
AS30	0.11	0.349	0.173	n.d.	n.d.	0.217	1.248	n.d.	0.083	1.209	0.002	0.538	n.q.	1.680	10.931	0.575	n.d.	10.931	0.575	0.133	29.080
AS31	0.13	0.353	0.250	0.614	0.435	0.355	2.273	n.d.	0.328	1.455	0.069	0.016	n.d.	3.284	22.589	1.663	n.d.	5.933	0.035	0.076	39.648
AS32	0.07	0.090	0.070	n.d.	n.d.	0.011	0.550	n.d.	0.012	0.475	n.d.	0.017	n.d.	0.615	4.495	0.185	n.d.	2.633	n.q.	n.q.	9.262
AS33	0.11	0.297	0.152	n.d.	n.d.	0.136	1.297	n.d.	0.066	1.169	n.q.	0.440	n.d.	0.800	12.022	0.442	n.d.	9.141	0.120	0.097	26.560
AS34	0.07	0.057	0.074	n.d.	n.d.	0.045	0.649	n.d.	0.179	0.607	n.d.	0.060	n.d.	0.999	6.702	0.292	n.d.	5.327	n.q.	0.050	15.042
AS35	0.09	0.464	0.226	n.d.	n.d.	0.282	2.099	n.d.	0.332	1.330	0.046	0.024	0.023	2.334	21.590	1.036	n.d.	5.515	0.101	0.150	36.177
AS36	0.30	1.458	1.078	n.d.	n.d.	3.859	10.076	n.d.	1.661	7.403	1.057	0.183	1.016	12.326	93.943	5.391	n.d.	27.645	0.446	0.912	175.706

^1^ Abbreviations: n.d.: not detected; n.q.: not quantified. Other abbreviations of the compounds are explained in the text and in Table 3. ^2^ Sum of peaks 1, 2, 5, 6, 7, 8, 9, 10, 11, 12, 13, 14, 15, 17, 18, and 19.

**Table 3:** Identity, mass spectral data, and retention indices (experimental AI_exp_ and theoretical AI_th_ on HP-5MS column) of volatile compounds in the garlic ethyl acetate extracts and previous references on their occurrence in garlic.

Peak No.	Compound	Molecular Formula	M.W.	*m*/*z* (%)	AI_exp_	AI_th_	Identification
1	ethyl vinyl sulfide (EVS) [22]	C_4_H_8_S	88.2	88 (100), 87 (65), 60 (41), 59 (41), 71 (22), 69 (18), 58 (9), 89 (7), 55 (7), 70 (6)	<800	690 [22]	MS, AI
2	diallyl sulfide (DS) [22,23,24]	C_6_H_10_S	114.2	97 (100), 112 (42), 98 (8), 111 (7), 53 (6), 99 (5), 77 (5), 69 (4), 113 (3), 114 (2)	854	855 [23]	MS, AI
3	m-xylene	C_8_H_10_	106.2	91 (100), 106 (57), 105 (26), 77 (14), 97 (11), 79 (10), 51 (10), 103 (8), 81 (8), 92 (7)	866	861.5 [30]	MS, AI
4	o-xylene	C_8_H_10_	106.2	91 (100), 106 (52), 105 (20), 77 (12), 51 (9), 79 (8), 92 (7), 103 (7), 78 (6), 65 (6)	892	894 [31]	MS, AI
5	methyl allyl disulfide (MADS) [22,23,24,26,27,28]	C_4_H_8_S_2_	120.2	120 (100), 79 (13), 80 (9), 122 (9), 73 (9), 64 (8), 121 (6), 71 (5), 72 (4), 87 (3)	915	916 [23]	MS, AI
6	3H-1,2-dithiole [24,25]	C_3_H_4_S_2_	104.2	103 (100), 104 (61), 105 (11), 71 (9), 69 (7), 59 (7), 64 (6), 58 (6), 106 (5), 57 (3)	951	958.6 [30]	MS, AI
7	3-methyl-2-cyclopentene-1-thione [26]	C_6_H_8_S	112.2	79 (100), 112 (96), 97 (71), 77 (62), 85 (40), 84 (34), 111 (31) 67 (18) 58 (18), 78 (17)	1001	-	MS
8	4-methylcyclopenta-1,3-diene-1-thiol	C_6_H_8_S	112.2	79 (100), 77 (44), 85 (36), 97 (30), 112 (26), 111 (21), 71 (21), 80 (20), 84 (15), 53 (15)	1004	-	MS
9	diallyl disulfide (DDS) [22,23,24,25,26,27,28]	C_6_H_10_S_2_	146.3	81 (100), 146 (49), 105 (46), 113 (43), 73 (37), 79 (35), 85 (29), 103 (25), 71 (23), 72 (21)	1077	1080 [23]	MS, AI
10	3-dithiane or 3,4-dihydro-1,2-dithiin	C_4_H_6_S_2_	118.2	118 (100), 72 (78), 71 (51) 103 (27) 85 (23) 73 (13), 120 (10), 69 (7), 119 (7), 117 (5)	1094	-	MS
11	1-propenyl allyl disulfide [23,27]	C_6_H_10_S_2_	146.3	73 (100), 146 (80), 81 (75) 105 (46), 61 (38), 71 (38), 74 (30), 72 (28), 104 (20), 79 (16)	1097	1090 [27]	MS, AI
12	allyl methyl trisulfide (MATS) [22,23,24,26,27]	C_4_H_8_S_3_	152.3	87 (100), 73 (79), 111 (15), 79 (14), 88 (13), 64 (12), 152 (8), 71 (7), 89 (6, 75 (5)	1134	1138 [23]	MS, AI
13	unknown	C_5_H_10_S_2_	134.3	71 (100), 120 (99), 72 (90), 55 (24), 69 (13), 103 (8), 73 (8), 58 (6), 64 (6), 134 (1)	1170	-	MS
14	3-vinyl-4H-1,2-dithiin (3-VDT) [22,23,24,26,27,28,29]	C_6_H_8_S_2_	144.3	111 (100), 144 (85), 97 (66), 103 (55), 71 (47), 77 (44), 72 (40), 79 (38), 85 (16), 67 (12)	1185	1188 [23]	MS, AI
15	4H-1,2,3-trithiine [26]	C_3_H_4_S_3_	136.2	71 (100), 136 (89), 72 (49), 72 (49), 69 (20), 103 (17), 55 (14), 64 (13),70 (12), 138 (12), 140 (1)	1192	1201.5 [30]	MS, AI
16	1-dodecene	C_12_H_24_	168.3	55 (100), 69 (90), 70 (84), 56 (83), 71 (76), 83 (74), 97 (68), 57 (63), 84 (44), 72 (37), 111 (28), 168 (7)	1192	1192 [30]	MS, AI
17	2-vinyl-4H-1,3-dithiin (2-VDT) [22,23,24,26,27,28,29]	C_6_H_8_S_2_	144.3	72 (100), 71 (93), 144 (63), 111 (53), 97 (20), 103 (16), 73 (15), 79 (12), 69 (10), 85 (8)	1209	1214 [23]	MS, AI
18	2-vinyl-1,3-dithiane [27]	C_6_H_10_S_2_	146.3	146 (100), 74 (52), 117 (50), 72 (48), 73 (43), 71 (39), 103 (22), 113 (13), 85 (11), 148 (11)	1215	1208 [27]	MS, AI
19	diallyl trisulfide (DATS) [22,23,24,25,26,27,28,29]	C_6_H_10_S_3_	178.3	73 (100), 113 (87), 71 (19), 72 (16), 74 (12), 103 (12), 79 (10), 64 (9), 85 (9), 104 (9), 146 (8), 178 (7)	1296	1301 [23]	MS, AI

**Table 4:** Concentration of total sugars, total phenolics, and pyruvic acid, and evaluation of antioxidant capacity (FRAP and H_2_O_2_ scavenging activity) determined in the selected garlic genotypes.

				Antioxidant Activity	
	Total Sugars mg Sucrose Equivalents/100 g F.W.	Total Phenolics mg GA Equivalents/100 g F.W.	Pyruvic Acid μmol Sodium Pyruvate/100g F.W.	FRAP μmol FeSO_4_ Equivalents/100 g F.W.	H_2_O_2_ Scavenging g Ascorbic Acid Equivalents/100 g F.W.
AS01	233.4 ± 5.5	56.7 ± 3.5	789.3 ± 56.9	301.6 ± 25.5	3.3 ± 0.0
AS02	275.0 ± 43.9	16.0 ± 1.1	664.9 ± 6.4	158.6 ± 12.6	0.9 ± 0.1
AS04	348.4 ± 37.7	35.4 ± 3.1	5727.7 ± 156.4	262.3 ± 16.2	2.6 ± 0.0
AS05	211.9 ± 17.0	48.8 ± 2.6	4152.4 ± 107.5	328.5 ± 16.8	3.3 ± 0.0
AS06	404.3 ± 33.0	17.0 ± 1.4	927.8 ± 83.8	114.0 ± 10.7	1.2 ± 0.0
AS07	184.8 ± 22.5	12.3 ± 1.3	369.5 ± 46.5	106.5 ± 8.0	1.0 ± 0.2
AS08	758.1 ± 20.1	32.6 ± 3.3	4342.9 ± 104.3	280.0 ± 17.4	2.1 ± 0.0
AS09	97.3 ± 8.1	13.0 ± 1.0	494.4 ± 58.2	78.6 ± 2.7	3.3 ± 0.0
AS10	552.2 ± 30.9	43.7 ± 3.3	2070.9 ± 287.5	275.6 ± 21.3	3.9 ± 0.1
AS11	254.9 ± 37.5	11.7 ± 0.7	1675.2 ± 129.5	133.9 ± 10.2	1.7 ± 0.0
AS12	628.7 ± 72.1	37.1 ± 3.0	7246.7 ± 527.7	339.2 ± 9.8	1.9 ± 0.0
AS13	365.0 ± 15.1	33.6 ± 2.4	2397.9 ± 249.2	207.4 ± 10.5	2.9 ± 0.1
AS14	174.2 ± 19.4	30.3 ± 2.5	2283.8 ± 248.6	172.5 ± 2.4	1.1 ± 0.1
AS15	503.9 ± 84.6	63.7 ± 5.4	5647.5 ± 237.9	336.3 ± 15.5	3.4 ± 0.1
AS16	450.1 ± 39.6	48.1 ± 3.0	2989.3 ± 243.3	260.8 ± 21.1	4.7 ± 0.0
AS17	323.6 ± 15.5	51.6 ± 3.6	4881.7 ± 259.2	412.2 ± 32.7	3.4 ± 0.1
AS18	113.6 ± 11.5	29.9 ± 1.6	1451.7 ± 84.6	238.9 ± 20.8	1.6 ± 0.0
AS19	147.4 ± 16.0	25.8 ± 1.1	2548.8 ± 156.2	193.7 ± 3.6	2.0 ± 0.1
AS20	125.0 ± 7.0	40.6 ± 3.2	2144.3 ± 203.0	269.2 ± 12.5	2.6 ± 0.0
AS21	381.9 ± 47.8	32.1 ± 2.5	1136.7 ± 38.0	251.8 ± 22.0	1.7 ± 0.1
AS22	336.4 ± 25.9	18.7 ± 1.8	1403.6 ± 112.8	123.7 ± 10.3	0.8 ± 0.2
AS23	439.9 ±41.6	25.9 ± 2.7	1823.1 ± 213.8	182.5 ± 14.5	0.8 ± 0.0
AS24	298.2 ± 27.4	50.9 ± 3.8	3143.6 ± 147.6	260.2 ± 3.7	2.3 ± 0.7
AS25	404.2 ± 24.5	38.9 ± 3.0	4993.1 ± 105.1	270.8 ± 9.9	4.1 ± 0.0
AS26	291.7 ± 28.1	46.3 ± 4.0	3364.7 ± 300.1	308.1 ± 19.5	2.5 ± 0.3
AS27	355.1 ± 26.9	43.6 ± 2.3	3616.2 ± 259.4	353.6 ± 31.0	2.0 ± 0.1
AS28	231.7 ± 18.5	19.8 ± 1.8	1242.6 ± 110.9	147.9 ± 19.3	1.4 ± 0.1
AS30	534.5 ± 17.3	53.9 ± 3.9	6790.6 ± 255.8	320.2 ± 6.7	2.8 ± 0.2
AS31	943.0 ± 11.4	81.9 ± 6.5	3673.9 ± 278.5	705.3 ± 70.0	2.2 ± 0.1
AS32	447.0 ± 49.5	28.2 ± 2.2	1161.5 ± 48.1	198.5 ± 23.2	1.7 ± 0.0
AS33	131.7 ± 5.9	27.4 ± 2.6	1272.7 ± 120.8	180.9 ± 17.2	1.0 ± 0.03
AS34	701.3 ± 91.2	47.2 ± 3.9	1864.7 ± 142.6	265.2 ± 9.2	1.9 ± 0.0
AS35	335.1 ± 109.7	50.4 ± 4.6	3311.9 ± 163.7	280.2 ± 33.7	4.1 ± 0.0
AS36	597.3 ± 53.5	51.2 ± 4.4	7066.4 ± 251.4	285.6 ± 26.9	2.8 ± 0.1

**Table 5:** Correlation table of garlic polar ingredients and antioxidant properties of the 34 genotypes.

	Sugars	Phenolics	Pyruvic	FRAP	H_2_O_2_
**Sugars**	1				
**Phenolics**	0.427 *	1			
**Pyruvic**	0.476 *	0.660 **	1		
**FRAP**	0.468 **	0.880 **	0.764 **	1	
**H_2_O_2_**	0.191	0.690 **	0.521 **	0.599 **	1

* Correlation is significant at the 0.05 level (two-tailed). ** Correlation is significant at the 0.01 level (two-tailed).
